# Types of Azygos Distal Anterior Cerebral Artery Branching Patterns: Relevance in Aneurysmal Surgery

**DOI:** 10.7759/cureus.681

**Published:** 2016-07-11

**Authors:** Harnarayan Singh, Sivashanmugam Dhandapani, Suresh N Mathuriya

**Affiliations:** 1 Neurosurgery, Fortis Memorial Research Institute; 2 Department of Neurosurgery, Postgraduate Institute of Medical Education and Research, Chandigarh; 3 Department of Neurosciences, Medipulse Hospital

**Keywords:** azygos distal anterior cerebral artery, aneurysm, trifurcation, quadrifurcation

## Abstract

Azygos distal anterior cerebral artery (Az.DACA) is a rare anatomical variant. This variant has been found to be associated with aneurysms in a significant proportion of patients. We present two cases of Az.DACA aneurysms associated with this anatomical variant with different branching patterns and the corresponding technical difficulties in clipping such aneurysms. Aneurysms associated with Az.DACA present unique technical challenges in proportion to the number of branches arising near the neck and should be managed at high volume centres with the best of facilities.

## Introduction

Az.DACA is an unpaired median vessel formed by the fusion of bilateral A2 segments of anterior cerebral artery (ACA), first described as "arteria termatica" by Wilders [1]. The incidence of this variant has been quoted to be 0.5-5% [2]. Though rare, aneurysms are associated with this variant in 41-71% of cases [3]. This presents a unique surgical challenge due to a single artery perfusing both ACA territories, difficult anatomy, and higher hemodynamic stress.

We present two cases of aneurysms arising at the division of Az.DACA, the technical difficulties faced, and review of the literature.

## Case presentation

**Figure 1:**
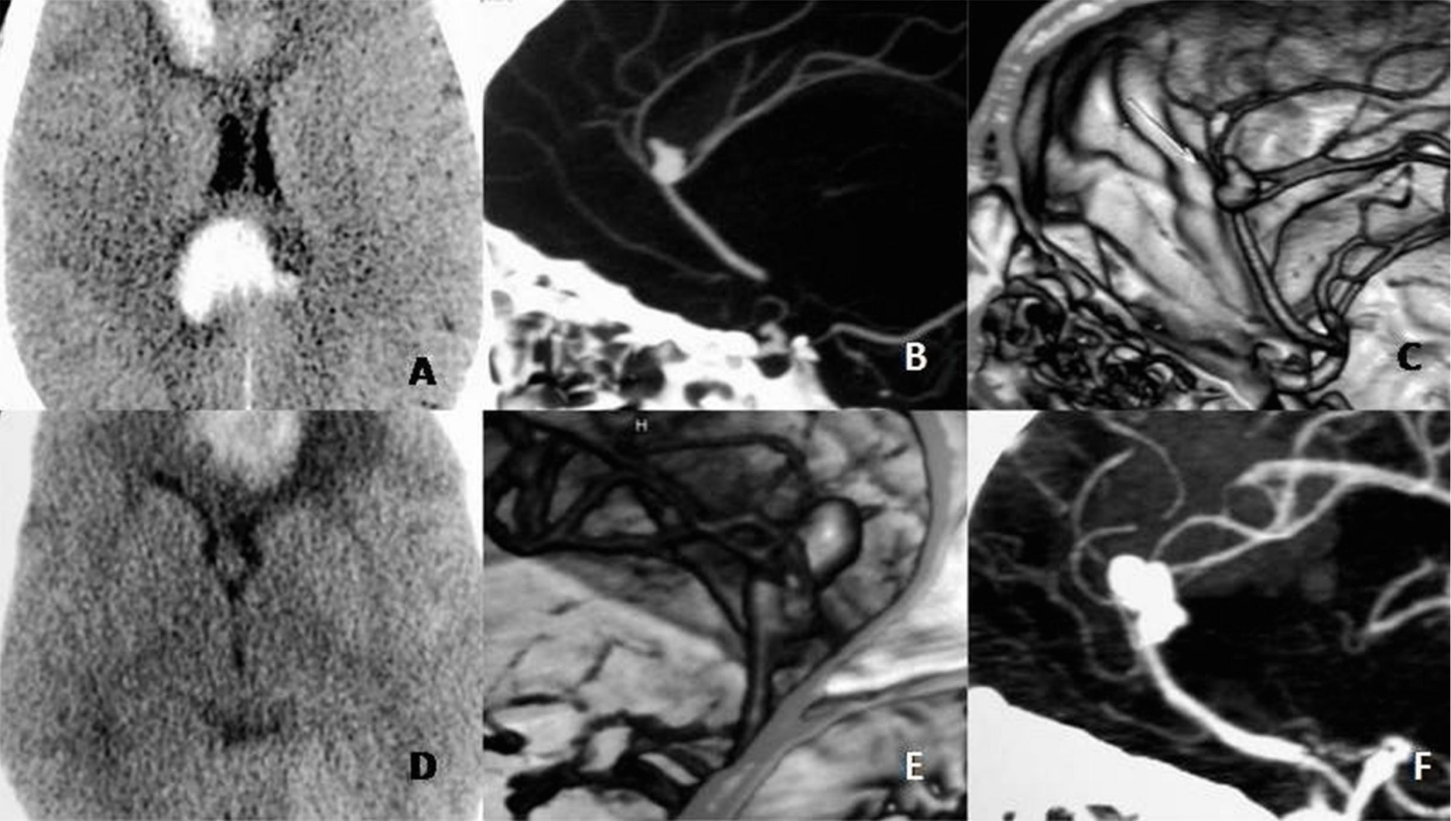
Preoperative Noncontrast CT and CT angiography images Images A-C (Case 1) - showing saccular aneurysm arising from an Azygos A2 ending in a quadrifurcation. Images D-F (Case 2) - showing Azygos A2 ending in a bifurcation, with close origins of distal branches that may give a pseudo-quadrifurcation pattern on angiography.

Case 1: A 35-year-old male presented with a history of sudden, severe headaches. It was followed by transient loss of consciousness. At presentation, he was conscious with no focal neurological deficits (H&H grade II). A computerized tomography (CT) scan revealed a dense clot in the anterior interhemispheric fissure (AIHF), corpus callosum, and intraventricular hemorrhage (IVH). A CT angiography (CTA) showed a single A2 trunk, quadrifurcating into two pericallosal arteries and two callosomarginal arteries, with a 1.2 x 1.1 x 7 mm aneurysm at the quadrifurcation. (Figure 1, A-C)

Case 2: A 55-year-old female had a sudden onset of a severe headache. She was conscious with no focal deficits at presentation. A CT scan revealed a thick clot in the AIHF. A CTA depicted an azygos A2, with an aneurysm at its bifurcation. The callosomarginal arteries were arising from the proximal 2-3 mm of the two divisions. (Figure 1, D-F)

Informed consent was obtained from the patients for this study.

In the first case, the aneurysm was approached through bifrontal craniotomy and basal interhemispheric corridor. The aneurysms were secured with a single permanent fenestrated right angle clip that took one of the two azygos A2 in the fenestration. An indocyanine angiography (ICG) revealed that the flow in one A2 was getting compromised while applying a tentative straight clip. Therefore, one A2 was taken into the fenestration of a right angled clip. There was no temporary clipping or intraoperative rupture. He underwent a bifrontal secondary decompressive craniotomy for refractory raised intracranial pressure. The patient had mild weakness of the lower limbs at the three-month follow-up. The weakness improved with hydration and there was no radiological vasospasm on CTA.

The second patient also underwent a bifrontal craniotomy and clipping of the aneurysms. Intraoperative patency of all branches was confirmed using ICG angiography. The aneurysms were secured with a single curved clip without temporary clipping or intraoperative rupture. Postoperatively, the patient developed transient monoparesis of the right lower limb that responded to hyperdynamic therapy. At the three-month follow-up, the patient made an excellent recovery in her motor deficit albeit with a mild deficit in bladder control.

## Discussion

Azygos variant of DACA is a rare entity caused by the unusual fusion of the normally paired A2 segments of ACA. This originates either from the medial branch of the olfactory artery at the initial 16 mm stage (day 40) of embryogenesis or the continuation of the median artery in the corpus callosum at the 20-24 mm stage (day 44). It can also be generated by a lack of development or regression of the ACA [3-5]. Az.DACA can be associated with developmental anomalies like fenestration, or hypoplasia of the A1 segment [3-4].

DACA aneurysms, which normally constitute around five percent of ruptured aneurysms, are noted in 41-71% in the presence of this variant [6-8]. There is a high incidence of non-saccular complex aneurysms at this location [8]. The branching pattern associated with this anomaly may vary, the most common being division into two pericallosal arteries. One case is reported in the literature with the azygos A2 trifurcating into the pericallosal arteries and one callosomarginal artery [9]. In Case 1 of our studies, there was a quadrifurcation pattern, with the A2 dividing in two pericallosals and two callosomarginals.

Baptista A.G. [10] divided abnormalities of the distal portion of the ACA into three groups: 1) single unpaired ACAs, in which a single ACA feeds into the medial surface of both cerebral hemispheres; 2) bihemispheric ACAs, in which there are two ACAs, but one is clearly dominant with branches extending into the contralateral hemispheres; 3) accessory ACAs, in which a third, or median artery, is distributed to either one or both hemispheres. Of these, the most important are the azygos variant [1]. The anatomy at this branching point is variable, with possible bifurcation, trifurcation or even quadrifurcation [8].

We would like to propose a further subtyping of the Az.DACA, depending on its division pattern. Type 1A, showing a bifurcation of Az.DACA, type 1B showing a trifurcation, and type 1C showing a quadrifurcation pattern of branching.

The most common site for these aneurysms is at the bifurcation and rarely at the proximal end mimicking an anterior communicating artery (ACOM) aneurysm [3]. The increased incidence of aneurysms associated with Az.DACA is believed to be due to increased blood flow velocities, and hemodynamic stress [2, 4, 8]. The aneurysms may accompany the formation of a congenitally anomalous artery. However, Wojciech, et al. did not find increased flow velocities in the azygos A2 and believed that an important role in their development is played by hemodynamic stress related to the bifurcation morphology of the distal end of the azygos ACA [9].

Commonly, these aneurysms present as subarachnoid hemorrhage [1, 3, 9]. These are rare cases in which onset of acute akinetic mutism caused by enlargement of a giant aneurysm resulting from thrombus formation within the aneurysmal sac have been reported [11].

The clinical significance of the azygos artery is vital. It is intimately associated with the formation of aneurysms and development of possible neurological deficits. These deficiencies are caused by ischemia in both hemispheres brought on by arterial damage or occlusion during an operation for an aneurysm [1]. The tolerance time for temporary occlusion of the distal ACA is obscure, particularly for the azygos artery. The tolerance time for the ACA might be similar to that of the middle cerebral artery (MCA), because flow rates in the distal ACA (120 ml/min) are comparable to those of the MCA [1]. Temporary clipping in the proximal A2 is an essential adjunct intervention and should be performed vigilantly in order to avoid ischemic damage in both hemispheres resulting from impaired perfusion [1].

The azygos artery is frequently associated with other malformations of the central nervous system (CNS), such as porencephalic cysts, agenesis of the corpus callosum, hydranencephaly, saccular aneurysms, and arteriovenous malformation (AVM). Among them, the incidence of aneurysms is about 13-71% [1]. Both the cases under study did not have any associated malformations.

The technical expertise required to handle azygos bifurcation aneurysm is more demanding in view of the complex branching patterns and complex aneurysm morphologies. Temporary clipping, tentative clipping, multiple clips, trapping, revascularization, and neck reconstruction may be required during surgery. The greater the number of branches arising from a single vessel in relation to the aneurysms, the greater the difficulty in successful clipping. Hence, the aneurysms associated with a quadrifurcation pattern of branching are the most difficult to treat. 

ICG angiography and intraoperative Doppler are extremely handy while clipping these aneurysms to ascertain the patency of distal circulation after clipping [8].

## Conclusions

Aneurysms of the Az.DACA are rare and present unique technical challenges. Hence, they should be dealt with by experienced cerebrovascular experts at high volume centers.
